# Aortic dissection, a diagnostic challenge

**DOI:** 10.1007/s12471-017-1026-8

**Published:** 2017-07-25

**Authors:** W. W. Jansen Klomp, A. P. Nierich, A. W. J. van ’t Hof

**Affiliations:** 1 Department of Cardiology, Isala, Zwolle, The Netherlands; 2 Department of (Thoracic) Anaesthesia and Intensive Care, Isala, Zwolle, The Netherlands

To the Editor,

We recently published a study in which we showed that acute aortic dissection (AAD) is often initially missed, which was contributable to the absence of a typical clinical profile [1]. In addition to our findings, Siniorakis and colleagues stress the importance of fever as an often overlooked sign of AAD.

The body temperature on admission was not incorporated in our database, as such we cannot give information on this topic with regards to our cohort. Following the scarce literature we agree, however, with the points stressed by Siniorakis. Indeed, data from the International Registry of Acute Aortic Dissections (IRAD) have shown that fever was an independent predictor of a delayed diagnosis, with a median time from admission to diagnosis of 32 vs. 4 h [2]. Although fever is usually an inflammatory response to the dissection itself, it may also precede AAD in the rare case of complicated aortitis.

It is important for physicians in the emergency care department to be aware that fever may be a sign of AAD, which should not distract from the important diagnostic work-up for AAD.
